# Activities of Antioxidant Scavenger Enzymes (Superoxide Dismutase and Glutathione Peroxidase) in Erythrocytes in Adult Women With and Without Type II Diabetes

**DOI:** 10.1080/15438600490451659

**Published:** 2004

**Authors:** Eli Carmeli, Raymond Coleman, Yitshal N. Berner

**Affiliations:** 1 Physical Therapy Department Sackler Faculty of Medicine Tel Aviv University Ramat Aviv 69978 Israel; 2 Department of Anatomy and Cell Biology Bruce Rappaport Faculty of Medicine Technion-Israel Institute of Technology Haifa Israel; 3 Geriatric Medicine Meir Hospital Kfar Saba Tel Aviv University Sackler Medical School Israel

## Abstract

It is widely believed that oxidative stress plays an important role in the pathogenesis of type II diabetes. The present study was undertaken to examine the functioning of two antioxidant scavenger enzymes, superoxide dismutase (SOD) and glutathione peroxidase (GSH-Px), in erythrocytes in a population of healthy aging adult women compared with a similar population with type II diabetes. Blood samples were examined from 42 female adult healthy subjects at different ages and from 59 female patients with type II diabetes. A significant increase in SOD activities was correlated with aging in erythrocytes of the healthy control subjects (*r* = .550, *P* = .001); however, this correlation was not found in subjects with type II diabetes (*r* = .250, *P* < .07). A trend showing a reduction in glutathione peroxidase activities was demonstrated with aging (*r* = −.331, *P* = .228); however, this trend was not found in diabetic subjects (*r* = .031, *P* < .820). The results indicate a possible imbalance in the antioxidant system in erythrocytes of aging adult women, which is even more pronounced in cases of type II diabetes. This study may indicate possible therapeutic treatment or preventive measures to limit oxidative damage and reduce complications of diabetes.


*Experimental Diab. Res.*, 5:171–175, 2004
ISSN: 1543-8600 print / 1543-8619 online
DOI: 10.1080/15438600490451659

Taylor & Francis
Taylor & Francis Group

### Activities of Antioxidant Scavenger Enzymes (Superoxide Dismutase and Glutathione Peroxidase) in Erythrocytes in Adult Women With and Without Type II Diabetes

**Eli Carmeli,[1] Raymond Coleman,[2] and Yitshal N. Berner[3]**

[1]*Physical Therapy Department, Sackler Faculty of Medicine, Tel Aviv University, Israel*
[2]*Department of Anatomy and Cell Biology, Bruce Rappaport Faculty of Medicine, Technion-Israel Institute of Technology, Haifa, Israel*
[3]*Geriatric Medicine, Meir Hospital, Kfar Saba, Affiliated to Tel Aviv University Sackler Medical School, Israel*

**It is widely believed that oxidative stress plays an important role in the pathogenesis of type II diabetes. The present study was undertaken to examine the functioning of two antioxidant scavenger enzymes, superoxide dismutase (SOD) and glutathione peroxidase (GSH-Px), in erythrocytes in a population of healthy aging adult women compared with a similar population with type II diabetes. Blood samples were examined from 42 female adult healthy subjects at different ages and from 59 female patients with type II diabetes. A significant increase in SOD activities was correlated with aging in erythrocytes of the healthy control subjects ($r = .550$, $P = .001$); however, this correlation was not found in subjects with type II diabetes ($r = .250$, $P < .07$). A trend showing a reduction in glutathione peroxidase activities was demonstrated with aging ($r = -.331$, $P = .228$); however, this trend was not found in diabetic subjects ($r = .031$, $P < .820$). The results indicate a possible imbalance in the antioxidant system in erythrocytes of ageing adult women, which is even more pronounced in cases of type II diabetes. This study may indicate possible therapeutic treatment or preventive measures to limit oxidative damage and reduce complications of diabetes.**

**Keywords** Diabetes; Erythrocytes; Glutathione Peroxidase; Superoxide Dismutase

Age-related degenerative changes affect a wide range of biological tissues. Accelerated aging changes that contribute to early morbidity are common in type II diabetes mellitus (DM) [1]. Oxygen homeostasis (prooxidant and antioxidant) is of critical importance for maintaining tissue viability and function. Oxidative stress involves imbalance between production of reactive oxygen species (ROS) and their removal. Both humans and experimental animals show age-related increases of reactive oxygen species, accompanied by changes in the system of antioxidant (AO) scavengers [2]. Individuals with type II diabetes show increased generation of ROS, eliciting both oxidative stress and glycation [3, 4]. As a protective measure, many cells and tissues in diabetic patients and experimental diabetic animals stimulate production of antioxidant enzymes such as superoxide dismutase (SOD) and gluthathione peroxidase (GSH-Px) [5–9]. Changes in the antioxidant system differ in various tissues. High oxidative stress is common in organs and tissues with high metabolic and energy demands, including skeletal and heart muscle, liver, lymphatic organs, and blood cells. In diabetic subjects, SOD and GSH-Px activities in skeletal muscle,

This is an original research that has not been published or submitted elsewhere that investigated the correlation between SOD and GSH-Px in adult women with and without diabetes. Its findings were presented in the 24th ESPEN Conference, Glasgow, Scotland.

The authors thank Shiri Kaminetzky, MSc, Faculty of Agriculture, Department of Biochemistry Nutrition, Hebrew University, Rechovot. The study was supported in part by a Wolf Research Grant of the Israel Academy of Sciences, and by Anne and Eli Shapira Charitable Foundations, Portland, Oregon, USA.

Address correspondence to Eli Carmeli, Department of Physical Therapy, Sackler Faculty of Medicine, Tel Aviv University, Ramat Aviv 69978, Israel. E-mail: elie@post.tau.ac.il spleen, retina, and other tissues may be reduced [10], whereas in other tissues, including kidneys and thymus, AO activities are in fact increased. Changes in the functional capacities of erythrocytes (RBCs) in human type II diabetes with aging have proved ambiguous [11, 12]. The main aim of the present study was to examine the functioning of two AO scavenger enzymes, SOD and GSH-Px, in a population of healthy aging adults in comparison with a similar age-matched population with type II diabetes (non–insulin-dependent DM, NIDDM).

### EXPERIMENTAL

### Subjects and Methods

All participants in the study provided informed consent. There were two study groups: (a) A type II diabetic group with at least 3 years since onset of the condition, consisting of 59 women; no subject required insulin. Their age range from 35 to 80, mean age 68 ± 7.4 from the Kupat Holim B Diabetes Clinic, Ashdod, Israel; and (b) a control group of 42 healthy women volunteers (age range 35 to 82, mean age 69.5 ± 7.1) matched for age, body mass index (BMI), lipid values, blood pressure (BP) (Table 1), and smoking status recruited from the staff of the Harzfeld Geriatric Hospital, Jerusalem. Subjects with dyslipidemia and under hormone replacement therapy were excluded.

Two milliliters of blood were drawn from the cubital vein of subjects after an overnight fast and collected in tubes containing an anticlotting agent (KF-Natrium II, EDTA). To prepare the samples of erythrocytes, the blood was centrifuged at 1000 rpm for 7 minutes in a refrigerated centrifuge and the plasma and buffy coat discarded, followed by washing of erythrocytes in normal saline and further centrifugation for 10 minutes. Determination of erythrocyte SOD and GSH-Px activities was based on the method described by Paglia and Valentine [13]. Briefly, SOD estimation was based on the generation of superoxide radicals produced by xanthine and xanthine oxidase, which react with 2-(4-idophenel)-3-(4-nitrophenol)-5-phenyltetrazolium chloride to form a red formazan dye. SOD activity was then measured by the degree of inhibition of this reaction. GSH-Px activity was based on the ability of this enzyme to catalyze the oxidation of glutathione by cumen hydroperoxide. Kits from Sigma were used for the enzyme assays. In the presence of glutathione reductase and NADPH, the oxidized glutathione is immediately converted to the reduced form with a concomitant oxidation of NADPH to $NADP^+$. The decrease in absorbance at 340 nm was measured using a Spectronic 401 spectrophotometer.

Protein was determined in diluted aliquots of the tissue homogenates by the method of Bradford [14].

**TABLE 1**
Comparison of the healthy women parameters (mean ± SD)

| Variables | Diabetic group (n = 59) | Control group (n = 42) | *P* values |
|---|---|---|---|
| Age (years) | 68 ± 7.4 | 69.5 ± 7.1 | NS |
| BMI | | | NS |
| Cholesterol (total) | 208.9 ± 26.4 | 206.6 ± 27.9 | NS |
| Triglycerides | 125.5 ± 34 | 128 ± 31 | NS |
| SBPmax (mm Hg) | 181.5 ± 17.2 | 184.8 ± 19.3 | NS |
| DBPmax (mm Hg) | 84.6 ± 11.7 | 85.2 ± 14.4 | NS |
| Glycated hemoglobin[a] (mg/dL) | 139.25 ± 5.1 | 95.08 ± 4.6 | .001 |

$P < .05$. BMI = body mass index; SBPmax = maximal systolic blood pressure; DBPmax = maximal diastolic blood pressure.
[a]Fasting blood glucose in serum.

### Statistical Analysis

All statistical analyses were subjected to a 2-way analysis of variance (ANOVA) using SPSS version 11, with the 2 main factors being healthy and having diabetes type II. To test for significant differences between means, a Fisher's least significant difference test was used. $P < .05$ was accepted as significant.

### RESULTS

The results are presented in Table 2. There was significant differences ($P < .001$) in SOD activities between healthy women and diabetic women, in all age categories, with total mean SOD activities of 184 ± 17.3 and 118 ± 13.1 units/mg protein, respectively. This differences were mainly because the absence of the increment of SOD activity with age. SOD activity levels at different ages in healthy controls and the diabetic (NIDDM) women are shown in Figure 1. Increasing SOD activities in erythrocytes are correlated ($r$ = from .541 to .561) significantly ($P = .001$) with increasing age in the healthy control subjects, whereas such a correlation does not exist in subjects with NIDDM ($r$ = from .249 to .253, $P < .066$–.071). The activity of GSH-Px in healthy women was from 1.02 up to 1.58 units/mg protein (mean of 1.20 ± 0.13 units/mg protein) and from 0.63 to 1.35 units/mg protein (mean of 0.99 ± 0.11 units/mg protein) in diabetic women. Changes of GSH-Px activities in different age groups among healthy and NIDDM subjects are shown in Figure 2.

There was an age-related decrease in GSH-Px activities in the healthy controls ($r$ = from −.329 to −.335, $P < .2$); however, such a weak correlation was absent in the diabetic group ($r = .031$, $P < .8$). No correlation ($r = -.172$) was found between the above enzymes. The duration since the onset of the diabetes did not affect the activities of SOD or GSH-Px. No

**TABLE 2**
The values of SOD and GSH-Px and their correlation to age and diabetes

| Group age | No. | Age mean ± SD | SOD (U/mg protein) | *r* | *P* | GSH-Px (U/mg protein) | *r* | *P* |
|---|---|---|---|---|---|---|---|---|
| Healthy control | | | | | | | | |
| 30–40 | 7 | 34 ± 2.2 | 99 ± 13.1 | .556 | *.001 | 1.58 ± 0.16 | .331 | *.228 |
| 41–50 | 9 | 46 ± 3.1 | 101 ± 15.8 | .541 | .001 | 1.21 ± 0.15 | .329 | .219 |
| 51–60 | 9 | 56 ± 2.8 | 160 ± 19.7 | .553 | .001 | 1.08 ± 0.12 | .334 | .230 |
| 61–70 | 9 | 67 ± 1.9 | 278 ± 18.9 | .561 | .001 | 1.02 ± 0.11 | .332 | .227 |
| 71–80 | 8 | 74 ± 3.0 | 282 ± 19.2 | .558 | .001 | 1.15 ± 0.12 | .335 | .229 |
| Mean | | | 184 ± 17.3 | | | 1.20 ± 0.13 | | |
| Diabetes | | | | | | | | |
| 30–40 | 9 | 35 ± 3.2 | 86 ± 11.2 | .253 | .070 | 0.79 ± 0.1 | .031 | .821 |
| 41–50 | 12 | 44 ± 3.3 | 144 ± 12.4 | .256 | .069 | 0.63 ± 0.17 | .030 | .818 |
| 51–60 | 14 | 55 ± 4.1 | 89 ± 11.6 | .249 | .071 | 1.17 ± 0.1 | .033 | .835 |
| 61–70 | 10 | 66 ± 2.1 | 140 ± 14.7 | .251 | .066 | 1.35 ± 0.1 | .032 | .810 |
| 71–80 | 14 | 74 ± 2.8 | 131 ± 15.5 | .252 | .069 | 1.04 ± 0.1 | .030 | .842 |
| Mean | | | 118 ± 13.1 | | | 0.99 ± 0.11 | | |

*$P$ significant at $<.05$; $r$, correlation coefficient.

correlation between indices of glycemia (glycated hemoglobin) and levels of SOD and GSH-Px was found in diabetic people.

### DISCUSSION

The aim of this study was to determine the activity levels of two AO scavenger enzymes in erythrocytes of adult-onset diabetics at different ages in comparison with age-, BMI-, lipids-, and BP-matched healthy controls. Radicals are involved in diabetes and related to a malfunction of AOs. In healthy control subjects the levels of SOD activities in erythrocytes showed marked progressive increases with aging, especially after age 60, whereas in the diabetic individuals erythrocyte SOD activities were elevated to a much lesser degree with aging. On the other hand, the GSH-Px activity does not change with age but rather it is slightly decreased, whereas in aged NIDDM subjects the activity of GSH-Px is increased. The increase of GSH-Px activity in specific biological cells in DM has been previously reported [10]. The increased production of radicals in diabetes may be associated by the presence of transition metal related to glycated proteins. As suggested before [12], glucose can be broken down into its final products and reduce cellular oxygen, yielding free radicals. Thus, free radicals may be detoxified by AO enzymes such as SOD or GSH-Px [15].

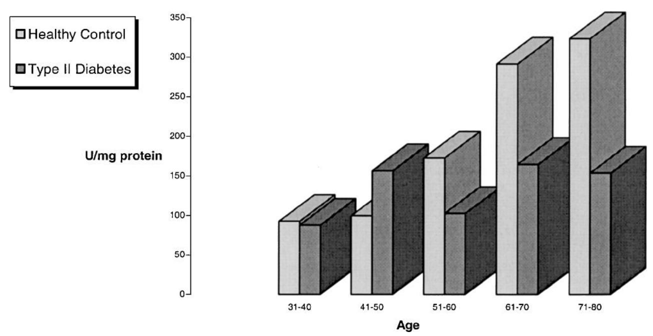

**FIGURE 1**
Superoxide dismutase (SOD) enzymatic activities with age in erythrocytes of healthy controls compared with type II diabetes.

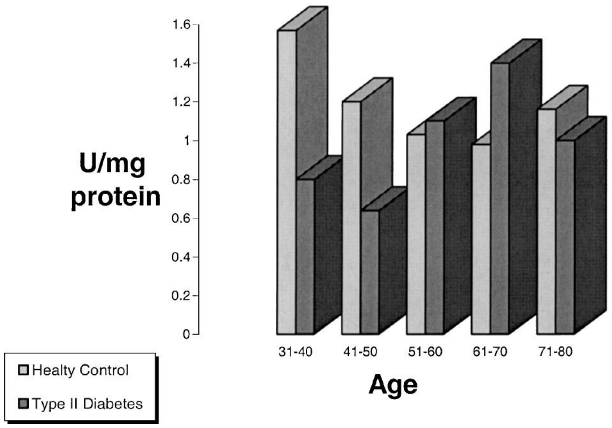

**FIGURE 2**
Glutathione peroxidase (GSH-Px) activities with age in erythrocytes in healthy controls compared with type II diabetics.

Two possible biochemical mechanisms can explain our findings: (a) GSH-Px is reduced by GSSG reductase, which is NAPDH dependent, and (b) increase in GSH-Px may be compensatory due to decrease in SOD activity in DM. Contrary to the findings of Kesavulu et al. [16], we found no change in GSH-Px between NIDDM and insulin-dependent DM (IDDM). In our study we found a temporary change in different enzymes activity.

Normally, erythrocytes contain enough AO enzymes such as SOD and GSH-Px. Yet, the AO enzyme activities in young and old diabetic individuals remained questionable. The total activity levels of AO (i.e., chelating agents) of ceruloplasmin, transferrin iron-binding capacity, RBC gluthathione, GSH-Px, and RBC SOD were lower in young DM patients [17]. In early (juvenile) DM in children, AO activity (GSH-Px, SOD) is increased, demonstrating increased oxidative activity. In aged DM subjects the AO activity is decreased [18].

Experimental diabetic rats show lower SOD in liver, kidney, heart, muscle, and erythrocytes [10]. Nishida et al. [19] demonstrated in experimental diabetes subjects a decrease of CuZn-SOD in retina in RBCs. However, it is a reversible phenomenon and within 3 days of insulin administration, the SOD levels returned to normal. Loven et al. [20] indicated a decrease of both CuZn-SOD and Mn-SOD in renal cortex. Also in this study the SOD levels returned to normal following 5 days of insulin treatment. Wohaieb et al. [21] also reported that CuZn-SOD changes are reversible in liver, pancreas, and kidney, but not in erythrocytes.

Saito et al. [22] and Bono et al. [11] demonstrated in humans no differences in SOD activities between NIDDM and non-NIDDM subjects. Selenium plays a role in reducing the oxidative stress associated with diabetes. A marked decreased in GSH-Px levels in DM subjects might be inhibited by selenium [23].

Our study did not find correlation between AO enzymes and the level of glucose following fasting. Kotake et al. [24] demonstrated that the decrease in activity of CuZn-SOD in erythrocytes of diabetics is consistent with glycosylation of the 3 active sites of CuZn-SOD, without any effect of hyperglycemia on the concentration of CuZn-SOD. One possible mechanism involved in the AO activity has to do with advanced glucosylation end products (AGEs) in diabetes and aging [24]. The increased nonenzymatic glycation has provided the basis of the linkage between DM and late complications in different tissues [4]. Thus, glycation reaction may proceed further to trigger oxidative stress, which leads to the accumulation of brown products, known as AGEs. Glycation occurs only in the presence of oxygen and the involvement of superoxide radicals and hydrogen peroxide [25]. Arai et al. [26] demonstrated that CuZn-SOD activity in erythrocytes of 31 humans was severely impaired in DM due to accumulation of carboxymethyl lysine, which is a major product of oxidative modification of glycated enzymes. In diabetes RBCs, glycosylated CuZn-SOD is higher. Incubation in vitro with glucose leads to glycosylation. The SOD active site contains histidine 72, whereas lysine is located at site 71, and glucose is connected to lysine residue to form Schiff-base response. The proximity of the sites may explain the inactivation of the SOD [27]. Antidiabetic treatment with *Capparis deciduas* decreased lipid peroxidation, yet altered SOD and catalase enzymes to reduce oxidative stress [28].

The intensification of GSH-Px and SOD activities in the present study may confirm the idea of a significant contribution of AOs to impaired homeostasis under diabetic conditions. Their activity is not necessarily age-related but rather glycemic-related response.

### CONCLUSIONS

The purpose of our study was to assess the parameters of two AO enzymes in RBCs of young and old NIIDM and nondiabetic women.

The activity level of superoxide dismutase and glutathione peroxidase in RBCs is correlated to age but only in nondiabetic women. The lack of correlation in NIDDM may indicates a possible imbalance in the AO system in erythrocytes of aging adult women, which is even more pronounced in cases of type II diabetes. This study may suggest a possible therapeutic treatment or preventive measures to limit oxidative damage and reduce complications of diabetes.

### REFERENCES

[1] Pietropaolo, M., and Le Roith, D. (2001) Pathogenesis of diabetes: Our current understanding. *Clin. Cornerstone,* **4,** 1–16.

[2] Carmeli, E., Lavian, G., and Reznick, A. Z. (2000) The role of antioxidant nutrition in exercise and aging. In: *Free Radicals in Exercise and Aging*, 1st ed., Edited by Radak, Z., pp. 73–115. Champaign, IL, Human Kinetics.

[3] Jennings, P. E., and Belch, J. J. (2000) Free radical scavenging activity of sulfonylureas: A clinical assessment of the effect of gliclazide. *Metab. Clin. Exp.,* **49(Suppl 1),** 23–26.

[4] Schleicher, E. D., Wagner, E., and Nerlich, A. G. (1997) Increased accumulation of the glycoxidation product N (epsilon) (carboxymethyl) lysine in human tissues in diabetes and aging. *J. Clin. Invest.,* **99,** 457–468.

[5] Sozmen, E. Y., Sozmen, B., Delen, Y., and Onat, T. (2001) Catalase/superoxide dismutase (SOD) and catalase/paraoxonase (PON) ratios may implicate poor glycemic control. *Arch. Med. Res.,* **32,** 283–287

[6] Pereira, B., Rosa, L. F., Safi, D. A., Bechara, E. J., and Curi, R. (1994) Superoxide dismutase, catalase and glutathione peroxidase activities in the lymphoid organs of diabetic rats. *J. Endocrinol.,* **142,** 161–165.

[7] Welsh, N., Margulis, B., Borg, L. A., Wiklund, H. J., Saldeen, J., Flodstrom, M., Mello, M. A., Andersson, A., Pipeleers, D. G., and Hellerstrom, C. (1995) Differences in the expression of heat-shock proteins and antioxidant enzymes between human and rodent pancreatic islets: Implications for the pathogenesis of insulin-dependent diabetes mellitus. *Mol. Med.* **1,** 806–820.

[8] Volkovova, K., Chorvathova, V., Jurcovicova, M., Koszeghyova, L., and Bobek, P. (1993) Antioxidative state of the myocardium and kidneys in acute diabetic rats. *Physiol. Res.,* **42,** 251–255.

[9] Gumieniczek, A., Hopkala, H., Wojtowicz, Z., and Nieradko, M. (2001) Differences in antioxidant status in skeletal muscle tissue in experimental diabetes. *Clin. Chim. Acta,* **314,** 39–45.

[10] Matkovics, B., Varga, S. I., Szabo, L., and Witas, H. (1982) The effect of diabetes on the activities of the peroxide metabolism enzymes. *Horm. Metab. Res.,* **14,** 77–79.

[11] Bono, A., Caimi, G., Catania, A., Sarno, A., and Pandolfo, L. (1987) Red cell peroxide metabolism in diabetes mellitus. *Horm. Metab. Res.,* **19,** 264–266.

[12] Sekeroglu, M. R., Sahin, H., Dulger, H., and Algun, E. (2000) The effect of dietary treatment on erythrocyte lipid peroxidation, superoxide dismutase, glutathione peroxidase, and serum lipid peroxidation in patients with type 2 diabetes mellitus. *Clin. Biochem.,* **33,** 669–674.

[13] Paglia, D. E., and Valentine, W. N. (1967) Studies on the quantitative and quantitative characterization of erythrocyte glutathione peroxidase. *J. Lab. Clin. Med.,* **70,** 158–168.

[14] Bradford, M. M. (1976) A rapid and sensitive method for the quantitation of microgram quantities of protein utilizing the principle of protein-dye binding. *Anal. Biochem.,* **72,** 248–254.

[15] Kamata, K., and Kobayashi, T. (1996) Changes in superoxide dismutase mRNA expression by streptozotocin-induced diabetes. *Br. J. Pharmacol.,* **119,** 583–589.

[16] Kesavulu, M. M., Kameswararao, B., Apparao, Ch., Kumar, E. G., and Harinarayan, C. V. (2002) Effect of omega-3 fatty acids on lipid peroxidation and antioxidant enzyme status in type 2 diabetic patients. *Diabetes Metab.,* **28,** 20–26.

[17] Telci, A., Cakatay, U., Satman, S., Satman, I., and Sivas, A. (2000) Oxidative protein damage on early stage type 1 diabetic patients. *Diabetes Res. Clin. Pract. Suppl.,* **50,** 213–223.

[18] Dominguez, C., Ruiz, E., Gussinye, M., and Carrascosa, A. (1999) Oxidative stress at onset and in early stages of type 1 diabetes in children and adolescents. *Diabetes Care,* **22,** 870–873.

[19] Nishida, T., Nakagawa, S., and Manabe, R. (1984) Superoxide dismutase activity in diabetic rat retina. *Jpn. J. Ophthalmol.,* **28,** 377–382.

[20] Loven, D. P., Schedl, H. P., Oberley, L. W., Wilson, H. D., Bruch, L., and Niehaus, C. L. (1982) Superoxide dismutase activity in the intestine of streptozotocin-diabetic rat. *Endocrinology,* **111,** 737–742.

[21] Wohaieb, S. A., and Godin, D. V. (1987) Alteration in free radical-tissue defense mechanisms in streptozocin-induced diabetes in rats. Effects of insulin treatment. *Diabetes,* **36,** 1014–1018.

[22] Saito, T., Ito, K., Kurasaki, M., Fujimoto, S., Kaji, H., and Saito K. (1982) Determination of true specific activity of superoxide dismutase in human erythrocytes. *Clin. Sci. (Colch.),* **63,** 251–255.

[23] Mukherjee, B., Anbazhagan, S., Roy, A., Ghosh, R., and Chatterjee, M. (1998) Novel implications of the potential role of selenium on antioxidant status in streptozotocin-induced diabetic mice. *Biomed. Pharmacother.,* **52,** 89–95.

[24] Kotake, M., Shinohara, R., Kato, K., Hayakawa, N., Hayashi, R., Uchimura, K., Makino, M., Nagata, M., Kakizawa, H., Nakagawa, H., Nagasaka, A., and Itoh, M. (1998) Reduction of activity, but not decrease in concentration of erythrocyte Cu,Zn-superoxide dismutase by hyperglycaemia in diabetic patients. *Diabet. Med.,* **15,** 668–671.

[25] Smith, P. R., and Thornalley, P. J. (1992) Mechanism of the degradation of non-enzymatically glycated proteins under physiological conditions. Studies with the model fructosamine, *N*(epsilon)-(1-deoxy-D-fructos-1-yl)hippuryl-lysine. *Eur. J. Biochem.,* **210,** 729–739.

[26] Arai, K., Iizuka, S., Tada, Y., Oikawa, K., and Taniguchi, N. (1987) Increase in the glucosylated form of erythrocyte Cu-Zn-superoxide dismutase in diabetes and close association of the nonenymatic glucosylation with the enzyme activity. *Biochim. Biophys. Acta,* **924,** 292–296.

[27] Oberley, L. W. (1988) Free radicals and diabetes. *Free Radic. Biol. Med.,* **5,** 113–124.

[28] Yadav, P., Sarkar, S., and Bhatnagar, D. (1997) Lipid peroxidation and antioxidant enzymes in erythrocytes and tissues in aged diabetic rats. *Indian J. Exp. Biol.,* **35,** 389–392.

